# Brain Spatial Genomics Atlases

**DOI:** 10.3390/genes17070745

**Published:** 2026-06-29

**Authors:** Alexander Hindeleh, Wei Xiong, Charles Wang

**Affiliations:** 1 Department of Pharmaceutical Sciences, School of Pharmacy and Pharmaceutical Sciences, University of California-Irvine, Irvine, CA 92697, USA; hindelehalex@gmail.com; 2 Department of Otolaryngology, Beijing Friendship Hospital, Beijing 100050, China; xw2208090209@163.com; 3 Center for Genomics, School of Medicine, Loma Linda University, Loma Linda, CA 92350, USA; 4 Department of Basic Sciences, School of Medicine, Loma Linda University, Loma Linda, CA 92350, USA

**Keywords:** spatial genomics, spatial transcriptomics, brain spatial genomics atlas, brain spatial transcriptomics atlas, single-cell sequencing

## Abstract

Recent advances in single-cell RNA sequencing (scRNA-seq) have transformed neuroscience research by enabling the identification of genes, cell types, and molecular pathways involved in brain development and function. However, scRNA-seq lacks spatial information regarding the anatomic location of gene expression. Emerging spatial genomics technologies, including MERFISH, CosMx, Stereo-seq, and Visium Spatial Gene Expression overcome this limitation by enabling transcriptomic and epigenomic profiling within intact tissue architecture. Integration of spatial genomics with scRNA-seq has revolutionized genomics and biomedical research by allowing gene expression to be mapped in situ at cellular and even subcellular resolution. These advances have facilitated the construction of brain spatial genomics atlases in several species, including mouse, human, non-human primate, and zebrafish. Spatial genomics technologies are particularly valuable for defining cellular heterogeneity across brain regions and characterizing the spatial organization of neuronal circuits when integrated with single-cell sequencing approaches. These reference atlases provide powerful resources for investigating brain development, function and disease, and for identifying region-specific molecular signatures associated with neurological disorders. Here, we review currently available brain spatial genomics atlases and the spatial genomics technologies used to generate these reference resources.

## 1. Introduction

Spatial genomics focuses on studying gene expression and genome organization in the context of spatial location within tissues or cells. Spatial transcriptomics (ST) enables simultaneously measurement of thousands of genes while preserving their spatial localization with tissues, in some case at single-cell resolution [1]. A brain spatial genomics atlas (e.g., Allen Mouse Brain Atlas [2]) maps gene expression across brain regions in situ—that is, within their anatomical context—often at single-cell or near-single-cell resolution to create a reference atlas. These atlases usually combine spatial transcriptomics, single-cell RNA sequencing (scRNA-seq), and advanced imaging techniques to create a comprehensive view of how genes are expressed in space and time within the brain. In this article, we review recent advances in spatial transcriptomic and epigenomic brain atlases across multiple species, including mouse, human, zebrafish, and non-human primate. We review current and emerging applications of spatial omics technologies, e.g., studying specific genes or pathways that are dysregulated in diseases and accelerating the identification of novel therapeutics for neurological disorders. Additionally, we discuss prospects and potential impacts of brain spatial genomics atlases.

## 2. Major Spatial Genomics Technologies Used in Brain Spatial Genomics Atlases

Spatial genomics technologies have advanced rapidly in recent years [3]. A comprehensive review of these technologies is beyond the scope of this article. Instead, we provide a high-level overview of the major spatial genomics platforms, particularly those used to construct brain spatial genomics atlases. Table 1 summarizes the key features of these technologies, including the developer, commercial platform, sequencing- or imaging-based approach, year of release, detection mechanism, major applications, advantages and limitations. We also provide reference to the original publications describing each technology or the corresponding company webpage.

**Table 1:** Major spatial genomics technologies and their applications, advantages, and limitations.

Company	Technology	Sequencing or Imaging Based?	Year Released	Detection Mechanisms	Applications	Species	Advantages	Limitations	Reference
10x Genomics (Pleasanton, CA, USA)	Visium v2	Sequencing	2019	mRNA capture via barcoded probes	Whole transcriptome in tissue	Human, mouse, rat, zebrafish, etc.	High throughput, broad coverage	No single-cell resolution (55 µm spots)	[4]
10x Genomics (Pleasanton, CA, USA)	Visium HD	Sequencing	2022	2 µm × 2 µm barcoded squares	Near single-cell resolution spatial profiling	Human, mouse	Finer resolution than Visium v2	Not true single-cell resolution, complex	[5]
10x Genomics (Pleasanton, CA, USA)	Visium HD 3’	Sequencing	2022	3’ poly(A) capture	3’ gene expression spatial profiling	Human, mouse, rat, NHP, zebrafish	High sensitivity	Only fresh frozen tissue, 3’ bias	[6]
10x Genomics (Pleasanton, CA, USA)	Xenium	Imaging	2023	RCA + cyclic fluorescent imaging	Subcellular, targeted transcriptomics	Human, mouse, rat, NHP	High-plex, subcellular data	Panel-based (max 5000 genes), time intensive	[7]
Nanostring/Bruker (Seattle, WA, USA)	GeoMX DSP	Sequencing	2019	UV-cleaved barcoded probes	Spatial profiling of RNA/proteins	Human, mouse, rat, NHP	Compatible with FFPE, RNA + protein	ROI-based, no single-cell	[8]
Nanostring/Bruker (Seattle, WA, USA)	CosMX SMI	Imaging	2022	Cyclic imaging with barcoded probes	Subcellular RNA + protein profiling	Human, mouse	High-plex, single-molecule	High cost, ~1500 gene limit	[9]
Vizgen (Cambridge, MA, USA)	MERSCOPE	Imaging	2021	MERFISH (error-robust FISH)	Subcellular, multiplexed expression	Human, mouse	Single-cell/subcellular resolution	Limited to pre-selected panels	[10]
BGI/STOmics (Shenzhen, China)	Stereo-seq	Sequencing	2021	DNA nanoballs on slides	Whole-transcriptome profiling	Human, mouse, zebrafish	High resolution, large area	Complex setup and analysis	[11]
Bio-Techne (ACD) (Minneapolis, MN, USA)	RNAscope	Imaging	2012	Chromogenic/fluorescent ISH	Targeted RNA detection	Human, mouse, rat	Simple, complements IHC	Very low plexity (2–12 targets)	[12]
Resolve Biosciences (Monheim am Rhein, Germany)	Molecular Cartography	Imaging	2021	smFISH with error correction	High-plex RNA spatial profiling	Human, mouse	Single-molecule, 100+ genes	Custom design, expensive	[13]
AtlasXOmics (New Haven, CT, USA)	DBit-Seq	Sequencing	2022	Microfluidics-based barcode tagging	Spatial co-transcriptome/proteome	Human, mouse	Multi-omics, microfluidic precision	FF tissue only, not single-cell	[14]
AtlasXOmics (New Haven, CT, USA)	Spatial CUT&Tag	Sequencing	2022	Antibody + A-Tn5 + microfluidic barcode	Spatial epigenomic profiling	Human, mouse	Histone modification mapping	Needs fresh frozen, antibody-optimized	[15]
AtlasXOmics (New Haven, CT, USA)	Spatial ATAC-Seq	Sequencing	2022	Tn5-based barcoding of open chromatin	Chromatin accessibility mapping	Human, mouse	Epigenomic context spatially	No single-cell resolution, 50 µm	[16]

## 3. Mouse Brain Spatial Transcriptomic Atlas

In 2006, the Allen Institute for Brain Sciences released the Allen Mouse Brain Gene Expression Atlas, a first of its kind, detailed spatial atlas of all protein-coding genes across all mouse brain regions [2]. Through an extensive series of in situ hybridization (ISH) experiments, the investigators were able to map out over 20,000 genes in the mouse brain, providing the most substantial gene expression map. Although many parts of their experimental pipeline were automated, this atlas took several years to complete, as every gene required a separate ISH experiment. Nonetheless, the scientific community recognized the profound impact of this open-source atlas, and its usefulness in identifying new cell types and advancing neurological disease research.

In following years, scientists continued working towards speeding up the long processing time required for all these separate ISH experiments. There was a need for faster, higher-throughput methods, which brought rise to MERFISH (multiplexed error-robust fluorescence in situ hybridization) in 2015 [10]. MERFISH is an imaging-based spatial transcriptomics method that allows for simultaneous imaging of hundreds of genes in situ through a predefined gene panel. This method uses unique barcodes that are assigned to RNA species of interest, which then go through multiple rounds of fluorescent imaging to show the barcode present at specific locations in the tissue. This method was extremely innovative upon its release, as it featured an extensive error correction method that increased the accuracy of imaging-based ST greatly [10] (see Table 1).

In a landmark study, Zhang et al. used MERFISH coupled with scRNA-seq and single-nucleus RNA sequencing (snRNA-seq), to generate a spatially resolved cell atlas of the mouse primary motor cortex, a brain region responsible for motor control and movement. They profiled 300,000 cells and identified 95 distinct clusters, including both neuronal and non-neuronal cell types [1]. By combining MERFISH with the retrograde tracing tool cholera toxin subunit B, the authors determined the specific projection patterns of intratelencephalic neurons, which allows for high-resolution gene expression capture and spatial localization of primary motor cortex cell types [1]. This spatial atlas is key for further studies on cell types in the primary motor cortex, as its spatial specificity can hold valuable insights for understanding the specific functions of neurons in the motor cortex. It can be used by researchers interested in movement disorders, such as Parkinson’s disease, to compare the spatial gene expression profiles of healthy and motor-deficient mice.

Additionally, using MERFISH integrated with scRNA-seq, Yao et al. constructed an extensive, high-resolution spatial transcriptomic atlas for a whole mouse brain consisting of approximately 4.3 million cells (spatial dataset) plus scRNA-seq transcriptomic dataset from ~4.0 million brain cells. They found that non-neuronal subclasses are more broadly distributed amongst brain regions, unlike neuronal subclasses that are confined to certain regions [17]. They analyzed the neuronal and non-neuronal cell types across the brain and found a high degree of correspondence between transcriptomic identity and spatial specificity for each cell type [16]. Through the use of MERFISH, this whole mouse brain atlas establishes a benchmark reference atlas and is a foundational resource for integrative investigations of cellular and circuit function, development and evolution of the mammalian brain [17].

As the value of these reference atlases and spatial omics technologies were recognized by scientific community, many other imaging-based methods began to emerge over the years, including STARmap PLUS, which was demonstrated in the work of Shi et al. The authors constructed a spatial atlas of the mouse central nervous system at molecular resolution, targeting over 106 brain regions, and detecting a panel of 1022 genes [18]. STARmap PLUS uses specialized probes that convert RNA molecules into DNA amplicons with barcodes unique to specific genes, which are then visualized through multiple rounds of in situ sequencing within specialized tissue hydrogels. Although this method does not achieve the same high gene detection throughput as MERFISH, it offers significantly higher resolution by combining imaging with in situ sequencing directly within intact tissue. They integrated this method with the use of an adeno-associated virus to allow for spatial mapping of the central nervous system and the identification of key cell types in the brain, further validating the integration of spatial transcriptomics with other methods for cell-type identification [18]. There was a strong correlation between the tissue regions identified via molecular biomarkers and those outlined by anatomical dissection regions. Of significant importance were molecular markers for the brain regions such as the thalamus, striatum, olfactory bulb, and the cerebral cortex, all of which showed congruence on the markers identified by Shi’s spatial atlas and the Allen Institute’s in situ hybridization database [18]. Imaging-based ST methods were critical in advancing the field of spatial biology early on, but they suffer from some detection bias due to the need for predetermined gene panels. A more recently developed method from 10x Genomics (Pleasanton, CA, USA), Visium, is a sequencing-based spatial transcriptomics method that counteracts this shortcoming. In Visium, brains are sectioned onto a specialized slide with capture areas, containing nearly 5000 barcodes each [4]. This method, released in 2019, transformed the landscape of ST technologies through its whole transcriptome coverage, meaning it could detect all mRNA in a tissue section. Visium can detect 10 to 12 times more genes than methods like MERFISH, CosMX, and STARmap Plus. This allows for more unbiased discovery of gene expression differences in tissue sections at the cost of offering lower resolution compared to the imaging-based methods previously mentioned (Table 1).

Several studies have shown the effectiveness of Visium in delineating disease-specific gene expression differences. Using 10x Visium spatial transcriptomics for analysis of the prefrontal cortex of an alcohol-dependent mouse model, Salem et al. identified a wide array of genes with positive implications for alcohol dependence [19] and identified the specific brain regions that had the highest correlation with this dependence They showed nearly 2 dozen brain regions where the Visium spatial gene expressions [19] were highly concordant with the brain spatial transcriptomic data from the Allen Brain Atlas [2].

In addition, using the Visium spatial transcriptomic technique, Di Marco et al. constructed a spatial transcriptomic map for the embryonic mouse brain [20] in which they obtained the mouse brains at embryonic day 13.5, aimed to examine the spatial gene expression landscape at neurogenesis, which is a key developmental stage in the formation of neurons [21]. Neurogenesis has been shown to impact the substantial brain functions, such as memory formation in the hippocampus [22]. Di Marco et al. study utilized coronal sections at the lateral ganglionic eminence level which allowed for characterization of GABAergic neurons, alongside staining for mitotic and radial glia cells. Their study produced an extensive dataset highlighting neurogenic origins for many neuron types, including GABAergic and glutamatergic neurons. Integrated with a scRNA-seq dataset [23], their spatiotemporal map deciphered the distribution of differentially expressed genes throughout brain regions, which helped determine if certain genes were strictly expressed in certain subregions or scattered across different sections of the brain during the embryonic developmental stage [20]. Sequencing-based spatial transcriptomics methods have emerged as an incredibly useful tool for delineating spatial gene expression in neurodevelopment and neurological disease models. The reference atlases created by these methods are crucial for understanding normal and abnormal brain development, as well as in-depth studies of cellular and molecular mechanisms underlying neurodevelopment in several brain regions.

## 4. Mouse Brain Spatial Epigenomic Atlases

As spatial transcriptomic brain atlases have emerged recently, scientists have acknowledged the need for more extensive atlases of the brain epigenome. The epigenome, which is the complete set of chemical marks on DNA and associated proteins that regulate gene expression, is a key aspect to consider when constructing an atlas of mouse brain cell types [24]. These chemical modifications modulate gene expression by influencing the proximity of genomic regions to RNA polymerases and associated transcription factors. The epigenome is commonly studied via bisulfite sequencing to examine methylated cytosines, or via transposase-accessible chromatin with sequencing (ATAC) to survey sites open for transcription factor binding. Single-cell epigenomics paved the way for studying chromatin accessibility and differential methylation amongst brain cell types [24] and aided in determining structure-function relationships [25]. Now, the emerging field of spatial epigenomics aims to profile epigenomic changes such as DNA methylation, chromatin conformation and accessibility in a spatial context, accounting for these changes in situ. It’s a relatively new field compared to spatial transcriptomics but holds great potential at delineating the dynamic epigenome’s role in disease progression, behavioral changes, and neuronal function. Although it faces limitations in resolution, sensitivity to tissue processing, and sparse signals, scientists are quickly working to produce high-impact spatial epigenomic atlases to complement existing transcriptomic atlases.

DNA methylation, a hallmark of epigenomic regulation, is an epigenetic modification in which a methyl group is added to the cytosine ring at the 5-carbon position, typically in CpG dinucleotides [26]. This modification can suppress gene expression by blocking transcription factor binding at promoter regions, or by promoting chromatin condensation, thereby limiting the access of transcriptional complexes to DNA. Hypo- or hyper-methylation of specific genes can carry severe pathological consequences. For example, in Alzheimer’s disease, hypermethylation of the gene *PIN1* (peptidoprolyl cis/trans isomerase, NIMA-interacting 1 gene) is an important biomarker to distinguish Alzheimer’s from frontotemporal dementia [27]. The study of whole-genome methylation, known as methylomics, has emerged as a hallmark of gene regulation, offering new insights to the molecular mechanisms of many disease processes, particularly neurological disorders. To assess the spatial methylation diversity across the brain, Liu et al. constructed a DNA methylation atlas of the mouse brain at single-cell resolution where they used a combination of single-nucleus methylome sequencing (snmC-seq2), single-nucleus assay for transposase-accessible chromatin using sequencing (snATAC-seq), and single-nucleus methylation and chromosome conformation capture sequencing (sn-m3C-seq) at single-cell base-resolution [28]. These cutting-edge technologies allowed them to define the methylome, chromatin conformation, and the genome-wide accessible chromatin in adult postnatal day 56 (P56) mouse brains, enabling them to identify the transcription factor genes with differential methylation in spatial context within cortical excitatory neurons following integration with an artificial neural network (ANN) [28]. Use of the ANN predictive model revealed extreme spatial diversity within the neuron methylome in the mouse brain. It correctly predicted spatial information for most cell subtypes within the brain-dissection information, while providing data on the exact cortical layers [28]. The model was unable to differentiate between 5-methylcytosine and 5-hydroxymethylcytosine, two distinct epigenetic modifications with different roles in gene regulation. Further studies will be needed to spatially profile these modifications individually and investigate their contributions to epigenetic regulation in various brain regions. Nonetheless, this study demonstrated the possibility of studying chromatin characteristics within a spatial context, and the role of artificial networks in processing spatial information regarding brain methylome data. Moving forward, the methods described in this study can be applied to further studies on the dynamic epigenomic landscape of the brain following certain treatments or disease progressions.

As an accompanying study to their mouse developmental P56 brain spatial methylation atlas, Liu et al. constructed a single-cell DNA methylome and 3D multi-omic atlas of the adult mouse brain which demonstrated robust spatial diversity amongst the mouse brain, particularly in methylation patterns [29]. They used snmC-seq3 and snm3C-seq technologies to investigate hundreds of thousands of methylations and chromatin conformation-methylation joint profiles from over a hundred brain regions [29]. Coupled with MERFISH spatial transcriptomic profiling, they cross-referenced the datasets to demonstrate that epigenetic regulation is strongly correlated with the spatial transcriptomic data. The gene Rasgrf2 (Ras protein specific guanine nucleotide releasing factor 2), for instance, was differentially expressed and differentially methylated between different cortical layers [29]. Since cytosine DNA methylation is an important indicator for mouse behavioral studies, understanding spatial epigenomics in the dynamic mouse brain may shed some new lights on brain disorders, like attention-deficit/hyperactivity disorder (ADHD) [30] or anxiety disorders [31].

A notable limitation of spatial methylomics approaches, especially, is that spatial organization in the brain is often inferred rather than directly measured. The above studies by Liu. et al. used single-cell methylome profiles coupled with computational analyses to decipher spatial patterns [28,29]. Their first study used an artificial neural network to predict spatial organization, and the accompanying one integrated a spatial transcriptomics dataset to estimate spatial location. Therefore, the resulting spatial atlases are dependent on the accuracy of computational prediction models and cross-modal integration, which could influence biological interpretation. Additionally, single-cell methylome data are inherently sparse, as only a subset of methylated cytosines is captured in each cell, increasing reliance on computational methods for data interpretation.

Presently, there remains a need for more direct spatial methylation profiling methods to resolve methylation states in situ and construct more reference atlases. Overall, the current work on spatial methylomics demonstrates the impact of spatial epigenomic atlases in providing important context regarding gene regulatory mechanisms and also complementing transcriptomic data. In the future, researchers can leverage more comprehensive spatial epigenomic atlases to identify regulatory mechanisms underlying observed gene expression patterns.

## 5. Spatial Chromatin Accessibility and Histone Spatial Multiomic Profiling in the Developing Mouse Brain

Another component of epigenomics is chromatin accessibility, which describes the ability of macromolecules such as RNA polymerases and transcription factors to bind to DNA within chromatin, and is determined by the organization of chromatin-binding factors and nucleosomes that may block or expose DNA [32]. When chromatin is in a closed state, transcription factors are unable to bind to DNA and initiate transcription, which can lead to altered gene expression and has been associated with abnormal physiological and disease states. Chromatin accessibility is typically measured through ATAC-seq (assay for transposase-accessible chromatin using sequencing), or its single-cell/nucleus versions (scATAC-seq/snATAC-seq). However, these methods lack spatial context of chromatin accessibility. Spatial ATAC-seq is a newly developed method for profiling chromatin accessibility in a spatial context, enabling the construction of spatial epigenomic brain atlases [16]. This method provides spatial context to traditional chromatin accessibility assays (Table 1), like scATAC-seq, that suffer from a lack of spatial information and uneven selection of cell types due to the tissue dissociation procedure. Using spatial ATAC-seq by combining in situ Tn5 transposition chemistry and microfluidic deterministic barcoding system, Deng et al. produced high-resolution spatial chromatin accessibility information on brain tissues from mouse and human embryos [16]. Through integration with scATAC-seq for cell type identification, they showed distinct differences across 2 different developmental stages, E11 and E13, in the mouse brain. Combined with scRNA-seq, the capability of determining chromatin accessibility with spatial context will advance our study of the brain. It will allow for analysis of chromatin accessibility and its role in specific structure-function relationships within the brain, since specific cell types are often localized within one or multiple regions.

Furthermore, Zhang et al. developed a spatial CUT&TAG-RNA-seq as a method for layered, spatial multiomic co-profiling of histone modifications and whole transcriptome from embryonic and juvenile mouse brain, as well as adult human brain [33]. Single-cell CUT&TAG has already been used for determination of histone modifications such as H3K4me3, alongside many promoters and enhancers [34]. The spatial CUT&TAG-RNA-seq uses the same Tn5 transposition process as spatial ATAC-seq [16], but with the addition of an antibody targeted to the desired histone modification (Table 1). They assessed the spatial locations of three different histone modifications in tissue sections of postnatal days 21 and 22 mouse brains, and found dozens of distinct clusters for H3k27me3, H3k27ac, and H3K4me3, reflecting spatially resolved chromatin states that could correspond to cell-type-specific regulatory mechanisms and regional differences in gene activation or repression [33]. The integration between spatial CUT&TAG-RNA-seq and single-cell CUT&TAG data showed high concordance between the two datasets, validating the use of the spatial modality in analyzing genome-wide histone modifications [33,34].

As these technologies are still developing, several limitations remain, including a lack of standardized protocols, benchmark datasets, and cross-platform validation. The current protocols are also sensitive to tissue preservation conditions, offering the best results with fresh-frozen tissue. While more recent studies have shown success in conducting spatial ATAC-seq with formalin-fixed paraffin-embedded tissue, these protocols are still being optimized, and further validation is required to enable reproducible workflows across laboratories. Spatial CUT&TAG-seq and spatial CUT&TAG-RNA-seq are currently only compatible with fresh-frozen tissue. Additionally, they do not consistently achieve true single-cell resolution; therefore, each spatial pixel may contain chromatin accessibility patterns from multiple cells. As a result, cell type-specific regulatory elements can be difficult to assign, and many rare cell populations may be masked by nearby abundant cell types. It also complicates the cell-type assignment process. To address this limitation, spatial chromatin accessibility datasets can be integrated with scATAC-seq and scRNA-seq reference atlases. The accuracy of these approaches, however, is dependent on the quality of the reference datasets and the performance of the computational integration and deconvolution processes. Nonetheless, these tools provide valuable insights and can be applied towards many sectors of neuroscience research. They can be used to identify key disease biomarkers and regulatory mechanisms and enable the development of comprehensive spatial epigenomic atlases of healthy and diseased brain tissues.

## 6. Human Brain Spatial Transcriptomics Atlases Across Different Developmental Stages

Although there have been far more examples of spatial transcriptomics being used to construct brain atlases for mouse, researchers have begun to apply these techniques to other species, including human. Compared with other species, human brain expresses messenger RNA (mRNA) transcripts in a large number and with much more complexity [35]. For instance, comparisons of human brain gene expression with both mouse [36] and non-human primates [37,38] demonstrated that most of the differentially expressed genes between the species are upregulated in human, but this phenomenon is not apparent in other tissues [39]. In addition, the human brain expresses ~ 85% of all genes encoded in the human genome at some point during development [40], which is greater than any other human tissue type. Presumably, this increased level of gene expression is responsible, at least in part, for the higher levels of neuronal activity and overall cognitive function in human. Thus, it seems impossible that mouse and other animal models can replicate human brain gene expression patterns with appropriate level of fidelity. Unfortunately, it is often difficult to obtain human brain tissue samples, as they are usually postmortem or archived tissue samples that may have suffered from RNA degradation over time. Table 2 summarizes some of the currently published human spatial brain atlases, describing the key applications and broader implications of the atlases.

As previously discussed, the human brain exhibits high cell-type diversity and more regionality than mouse, making atlas construction more computationally intensive and expensive due to the need for more brain sections and more biological replicates. Many single-cell studies have explored specific brain regions during human development, mostly the neocortex regions from postnatal period to maturity [41,42]. However, these studies often lack information on the first trimester of development, typically due to difficulties in obtaining early embryo specimen. Braun et al. constructed a comprehensive cell atlas of the first trimester developing human brain by performing scRNA-seq and spatial transcriptomics from 26 human brain specimens spanning 5 to 14 postconceptional weeks [43]. They identified 616 clusters and annotated them with data including class and subclass, spatial location, distribution across embryonic ages, and specific gene expression markers. They found that the major cell types in the brain such as neurons, astrocytes, and oligodendrocytes, all developed from region-specific radial glia and transitioned through intermediate cell states [43]. Intriguingly, when compared with the adult brain, region-specific glia showed similar patterns in the embryonic brain, which suggests that the highly regionalized human brain was differentiated from region-specific astrocytes and oligodendrocytes [43]. It is worth noting that although no spatial genomics techniques were used in their single-cell analysis of prenatal and postnatal human brain cortical samples obtained at different stages from the 2nd trimester to adult, Velmeshev et al. delineated the developmental programs of specific cortical cell types in human brain, including subtypes of excitatory neurons, interneurons, and glial cells [44]. Furthermore, their study identified the key transcriptional networks during cortical development, as well as gender-specific developmental changes, which are crucial for exploring developmental brain disorders, such as autism [44].

Li et al. constructed a spatiotemporal developmental atlas of human embryonic brain including different brain regions such as neocortex (Cor), subpallium (Sp), diencephalon (Dien), midbrain (Mid), and cerebellum (Cere) at multiple developmental time points from gestational week 6 (GW6) to GW23 using single-cell spatial enhanced resolution omics sequencing (scStereo-seq) [45]. They annotated neuronal progenitor cells (NPCs) into five subclasses including radial glial cells (RG), Cor-specific intermediate progenitor cells (IPC), Cere-specific granule cell precursor (GCP), precursor 1 (Prec1), and precursor 2 (Prec2) according to the cell type specific marker genes [45]. scStereo-seq builds upon the traditional Stereo-seq method of spatial barcoding with DNA nanoballs on a chip, with additional single-cell resolution through a single-cell extraction method [46]. Of the five NPC subtypes, RG emerged first and showed significant regional heterogeneity at this stage, whereas the transcription factor TFAP2C was specifically expressed in human cerebral cortex RG. They observed that the RG cells presented in each brain region with a spatial distribution characteristic [45]. Spatial visualization not only shows the spatial distribution of RG subtypes but also provides key clues for later neuronal region specialization. Combining scRNA-seq with scStereo-seq spatial transcriptomics, the temporal and spatial development trajectories of specific subpopulations of neurons in each region were calculated and reconstructed, illustrating spatiotemporal dynamics and regional diversity of human brain neuron subtypes [45].

Spatial transcriptomics can also be used to study the connections within different layers, cross-sectional relationships, in the same brain region [47]. Maynard et al. combined snRNA-seq with the high-throughput 10x Genomics Visium spatial transcriptomics technology to define the spatial topography of gene expression in the six-layered human dorsolateral prefrontal cortex (DLPFC) and illustrated that neurons from different cortical layers showed different patterns of gene expression as well as different morphological, physiological and connection patterns [48]. They identified several genes such as FABP7 (fatty acid binding protein 7 gene), ADCYAP1 (adenylyl cyclase activating protein 1 gene) and PVALB (parvalbumin gene) that were expressed in DLPFC, which were not classified by the Allen Brain Institute’s resources as being layer markers [48]. Through integrated analysis of neuropsychiatric gene sets, they found that layer-enriched genes in the human brain were associated with schizophrenia and autism spectrum disorders, indicating the value of spatial transcriptomics in studying brain diseases [43].

Huuki-Myers et al. also constructed a high-resolution, data-driven spatial atlas using snRNA-seq and the Visium platform to map gene expression in the human DLPFC [49]. They used 30 DLPFC tissue blocks derived from 10 donors to identify distinct spatial domains and cell-cell communication patterns, which were found to be crucial for understanding the brain’s structure and function. This atlas provides insights into the molecular mechanisms underlying brain disorders and anatomical context for cell-type–specific gene expression changes associated with neurodevelopmental disorders and psychiatric illness [49].

Most of the existing studies focused on the spatial transcriptomics of normal or developing brain tissue, and few explored the changes of spatial transcriptomics in the pathological state. This knowledge gap was remedied in a recent study, in which Zheng et al. generated an atlas integrating spatial transcriptomics and metabolomics from six patients with brain trauma using surgical samples [50]. They found that spatial transcriptomics could reveal distinct molecular regionalization of injured brains and helped identify distinctive cell clusters between the moderate and severe brain injuries, which showed a different distribution of microglia, NK cells, T cells, and oligodendrocytes [50]. Additionally, neuronal loss was spatially concordant with the lipid peroxidation, metabolic change and local gene expression in injured brain [50]. This study opens the door to future research on injured brain regions, revealing new pathological hallmarks through cell-type studies. Sankowski et al. applied multi-omics including scRNA-seq and spatial transcriptomics and generated a multiomic spatial landscape of innate immune compartments at the human CNS during fetal development, adulthood, and pathological processes, revealing the spatial multi-omics atlas of innate immune cells at the CNS interface [51].

Furthermore, the thalamus is the higher center of sensation, and it is the most important sensory conduction relay station. Conduction pathways from various sensations throughout the body (except olfaction) are relayed in the thalamus, which then projects to the cerebral cortex [52,53]. In an effort to decipher the molecular diversity and spatial organization of cell types in the developing human thalamus, Kim et al. constructed a spatiotemporal molecular dynamics map of developing human thalamus [54]. Their study indicated that during early pregnancy, neurogenesis occurred in the thalamus, generating both glutamatergic and GABAergic neurons. By the mid-pregnancy stage, glutamatergic neurons differentiated into two subtypes, while the number of GABAergic neurons in the thalamus significantly increased and began to distribute widely [54]. Furthermore, they observed spatial resolution patterns of glial cells and their occurrence and maturation over time, such as two subtypes of astrocytes, one enriched in the thalamus and the other in adjacent brain regions. These findings contribute to a better understanding of the evolutionary process of the human brain, especially enhancing our understanding of thalamus development and the complexity of the human brain at the cellular level. Overall, the construction of human brain spatial transcriptomic atlases is still developing, but the field is rapidly advancing and garnering strong interest from the scientific community.

## 7. Non-Human Primate Brain Spatial Transcriptomics Atlas

Compared to other species, *Macaques* are the closest model animals to humans in terms of higher cognitive and social behavior, with larger cortexes and more cell types [55]. The *Macaque* brain consists of 6 billion neurons, which are classified into hundreds of different types based on their molecular, morphological, or physiological characteristics, and distributed across hundreds of different brain regions [48,56]. They are interconnected to form complex and intricate neural circuits, supporting higher cognitive and social behaviors, with abnormalities in their cells and circuits leading to various brain disorders. Deciphering the cellular composition and spatial distribution patterns in the *Macaque* cerebral cortex is crucial for elucidating the organizational principles of primate brains, and they are often used to validate human brain spatial transcriptomic and single-cell sequencing atlases due to their high genetic similarity [57]. Table 2 describes the few current available *Macaque* spatial brain atlases, including the scientific questions addressed and the broader implications of the work for the scientific community. Unlike human brain atlas, however, there is a lack of *Macaque* single-cell RNA-seq atlases, leading to incomplete cell-type annotation and spatial pattern validation. It is also much harder to obtain samples due to ethical constraints, and generally, mostly postmortem samples are available which are more susceptible to RNA degradation. To fill this knowledge gap, Chen et al. used snRNA-seq and Stereo-seq and completed the first single-cell spatial atlas of the *Macaque* cerebral cortex [58]. They processed 67 fixed-frozen coronal sections of the *Macaque* claustrum derived from the left hemisphere of two animals. They found that a large number of excitatory neurons, inhibitory neurons, and non-neuronal cells exhibit distinct layer-specific and region-specific distributions in the cerebral cortex, and there is a significant correlation between the cellular composition of the visual and somatosensory systems and the hierarchical organization of brain regions, with brain regions at the same level often having similar cellular compositions [58]. A cross-species comparison of single-cell data from monkey brain with human and mouse brain revealed that some layer IV excitatory neurons were found only in primates and that such neuron-specific highly expressed genes are associated with human diseases [58]. This study provides the most comprehensive data on a layer-specific and region-specific distribution of cell types within the cortex, as well as gene expression characteristics, in the primate brain to date. It lays the molecular and cellular foundation for further investigation into the connections between various types of neurons and serves as an important data resource for subsequent related research [58].

By using samples from Rhesus monkeys, Micali et al. focused on the development of the entire telencephalon and the molecular processes that regulate the spatial distribution of nerve cells using scRNA-seq and 10x Visium spatial transcriptomics technologies [59]. Based on the deep sequencing of more than 700,000 single cells, their study distinguished a variety of cell subtypes involved in neurogenesis, including neural stem cells, excitatory and inhibitory neurons, glial cells, and non-neural cells. In addition, they identified progenitor cells early in telencephalic center development and used this to predict the gene regulatory network that coordinates the function of this cell pattern [59]. These resources could allow scientists to better study telencephalon development, brain evolution, and disease in primates and humans.

Lei et al. constructed a comprehensive atlas of the adult *Macaque* cortex at the single-cell level, utilizing advanced spatial transcriptomics and chromatin accessibility techniques [60]. They focused on three key cortical areas: the prefrontal cortex (PFC), primary motor cortex (M1), and primary visual cortex (V1) [60]. By integrating scATAC-seq and scRNA-seq data with the spatially enhanced resolution omics-sequencing (Stereo-seq), they mapped the regulatory landscapes and chromatin accessibility across these regions. Furthermore, they compared the trajectories of oligodendrocytes in adult *Macaques* and humans to determine the similarities and differences between the two species [60]. This mapping of genetic variants and disease-related gene perturbations to specific cell types in the *Macaque* cortex provides valuable insights into the potential impacts of these factors on human neurological diseases.

**Table 2:** Summary of the current brain spatial genomics atlases of human and non-human primate.

Authors	Species	Scientific Question Addressed	Main Findings	Broader Implications	Reference
Braun et al.	Human	How can we map brain cell types and gene expression trajectories during the first-trimester of human embryonic development?	Reconstructed neuronal and glial differentiation trajectories; Revealed extensive diversity among early glioblast populations.	Provided a spatially resolved, single-cell atlas of first-trimester cortical development for future therapeutic target identification, organoid model validation, and comparisons to diseased brain tissue.	[43]
Velmeshev et al.	Human	How do human cortical cell types develop from prenatal to postnatal stages, and how might they be linked to neurodevelopmental disease risk?	Identified cell-type-specific developmental trajectories and gene regulatory networks across prenatal and postnatal human cortex	Generated a comprehensive cellular reference atlas of the developing human cortex, allowing for developmental cell state mapping onto cortical structures and emerging spatial domains; Provides a rich resource for studying differences in prenatal and postnatal developmental processes.	[44]
Li et al.	Human	How can we use spatial transcriptomics to assess gene expression patterns across regions of the developing human brain?	Identified region-specific gene expression patterns and developmental trajectories across different brain structures at gestational weeks 6–23	Provided a reference for studying human brain development and neurodevelopmental disorders in the developing brain with regional specification across different stages of gestation.	[45]
Maynard et al.	Human	How is gene expression organized across different layers of the human DLPFC?	Characterized layer-specific gene expression, novel cortical markers, and disease-associated genes within the human DLPFC	Among the first studies to apply Visium spatial transcriptomics to human brain tissue, particularly the DLPFC, providing a spatial reference atlas for examining cortical layer organization and disease-associated molecular alterations	[48]
Huuki-Myers et al.	Human	How are cell types spatially organized throughout the human prefrontal cortex?	Identified spatially distinct neuronal and glial populations, refining cell-type annotations for rare and specialized populations	Enabled studies of spatially organized cellular diversity, disease processes, and molecular mechanisms in the human prefrontal cortex	[49]
Zheng et al.	Human	How do spatial gene expression patterns and metabolic states change following injury?	Integrated spatial transcriptomics and metabolomics to delineate metabolic heterogeneity in injured brain tissue, revealing region-specific molecular and metabolic adaptations following injury. Demonstrated strong concordance between transcriptomic and metabolomic spatial patterns.	Established a framework for combining spatial transcriptomic and metabolomic datasets to study clinically-relevant genes and pathways in human brain tissue.	[50]
Sankowski et al.	Human	How are innate immune cells spatially organized at the borders of the human central nervous system (CNS)?	Revealed region-specific immune states, macrophage populations, and distinct transcriptional networks in CNS border-associated tissues.	Established a spatial multi-omic reference atlas for human CNS border-associated immune niches, providing a rich resource for future studies of neuroimmune interactions and cellular mechanisms underlying neurological disease.	[51]
Kim et al.	Human	Can we study the thalamus, the higher center for sensation in the brain, at spatial resolution to identify region-specific molecular patterns?	Generated a spatially resolved atlas of the developing human thalamus, identifying molecularly distinct thalamic regions and differentiation trajectories of glutamatergic and GABAergic neuronal populations.	Defines the spatial organization of the developing human thalamus, enabling future studies of disrupted cell differentiation and disorders related to thalamocortical circuits.	[54]
Chen et al.	Non-human primate	How do spatial gene expression patterns define the cellular architecture and regional specialization of the *Macaque* cortex?	Mapped *Macaque* cortical cell-type organization at single-cell and spatial resolution, identifying laminar distributions, and spatial localization of neuronal and glial populations; Revealed *Macaque*-specific neuronal transcriptional signatures.	Established a cortical reference atlas for *Macaques,* allowing for future studies of brain architecture in normal and disease-associated states.	[58]
Micali et al.	Non-human primate	How does spatial regionalization of the developing *Macaque* telencephalon influence neural stem cell (NSC) fate specification?	Characterized molecular networks underlying telencephalic regional specification and NSC progression, identifying developmental trajectories in the telencephalon.	Provided more insight into the *Macaque* telencephalon and how NSCs diversify; Useful for future studies of telencephalic development and neurodevelopmental disorders.	[59]
Li et al.	Non-human primate	How are gene regulatory networks spatially organized across the adult *Macaque* cortex?	Provided a spatially resolved atlas of gene regulatory networks and cortical areas enriched for disease-associated genes.	Establishes a spatially resolved framework for associating cortical gene regulation to disease vulnerability in non-human primate models.	[60]

## 8. Brain Spatial Transcriptomics Atlas of Zebrafish

Spatial transcriptomics has been applied towards other species, such as zebrafish. Zebrafish are widely used model organisms, mostly for their transparent embryos and rapid reproduction rate, making them ideal for researching vertebrate development [61,62]. A recent study, using the Stereo-seq technology, identified key pathways involved in the embryogenesis of the zebrafish [63]. Their study filled many gaps in the knowledge about zebrafish gene expression and cellular localization by applying Stereo-seq to zebrafish embryos at six time points within the first 24 h post-fertilization (hpf). This study provided spatially resolved data for ligand-receptor pairs and aided in cell-type determination for zebrafish [63]. Most notably, the 19 spatial modules represented by the 24-hpf sections showed high concordance in their spatial distribution and their biological functions within each region, as shown by a gene ontology (GO) analysis. Then, they modeled embryogenic cell-state transitions using the Stereo-seq data integrated with the scRNA-seq data, to map the developmental trajectories of cell types [63]. They found that 2 clusters of neural stem cells (NSCs) were very consistent throughout the two datasets. Through further spatial subclustering, it was determined that these NSCs would differentiate into other cell types, like hindbrain NSCs, or primary motor neurons [63].

The study of neurodevelopment in the developing zebrafish embryo is impactful and validates the role of spatial transcriptomics in building cell type atlases of the central nervous system. A similar study was conducted by Holler et al. that used spatial transcriptomics and scRNA-seq to examine the spatio-temporal transcriptome during the first few hours of the zebrafish development [64]. Particularly, the authors implemented the tomo-seq method to measure RNA localization in one-cell stage zebrafish embryos with high spatial resolution. They used patterning genes, such as dazl, to examine patterns of localization. Furthermore, when performing spatial transcriptomic analysis on two closely related species, Xenopus laevis and Xenopus tropicalis, they found that the gene, dazl (deleted in azoospermia-like gene), showed a conserved role in the specification of germ cells [64]. This study further validates the high value of spatial integration alongside scRNA-seq, and its use in building cell type atlas for the study of development in zebrafish.

## 9. Prospects for Brain Spatial Genomics Atlases

Although brain spatial genomics atlases have been constructed for mice, humans, and several other species, spatial brain genomics reference maps remain unavailable for rats and many additional species. Ongoing efforts are focused on creating more comprehensive and integrated brain atlases, incorporating multiomic data sources, and developing new technologies capable of mapping the brain at increasingly higher spatial resolution. As spatial genomics technologies continue to advance, brain spatial genomics atlases are expected to become available for many additional species, including rats.

Advances in artificial intelligence (AI) and computational modeling are also expected to transform the field by providing new insights into brain organization and function [65,66]. For example, integrating spatial genomics with AI may enable to reconstruction of three-dimensional brain atlases, prediction of function from structure, and development of computational models of brain disorders grounded in molecular and cellular architecture. AI-based approaches are also increasingly important for spatial genomics analysis, including cell type deconvolution, clustering, cell-cell communication analysis, integration with scRNA-seq and other single-cell multi-omic datasets. Deep learning and machine learning methods are expected to play a central role in overcoming the computational challenges associated with increasingly large and complex spatial genomics datasets [66].

Through continued technological innovation and interdisciplinary collaboration, brain spatial genomics atlases will become powerful resources for understanding brain functions and molecular basis of neurological diseases including Alzheimer’s disease [67,68] and Parkinson’s disease [69].

## Data Availability

No new data were created or analyzed in this study.
